# 4400 Low CD4 nadir linked to widespread cortical thinning in adults with HIV

**DOI:** 10.1017/cts.2020.77

**Published:** 2020-07-29

**Authors:** Shiva Hassanzadeh-Behbahani, Kyle F. Shattuck, Margarita Bronshteyn, Matthew Dawson, Monica Diaz, Princy Kumar, David J. Moore, Ronald J. Ellis, Xiong Jiang

**Affiliations:** 1 Georgetown - Howard Universities

## Abstract

OBJECTIVES/GOALS: The history of immune suppression, especially CD4 nadir, has been shown to be a strong predictor of HIV-associated neurocognitive disorders (HAND). However, the potential mechanism of this association is not well understood. This study examined the relationship between CD4 nadir and brain atrophy. METHODS/STUDY POPULATION: Fifty-nine people with HIV participated in the cross-sectional study (mean age, 56.5 ± 5.8; age range, 41-69; 15 females; 46 African-Americans). High resolution structural MRI images were obtained using a 3T Siemens scanner. From a comprehensive 7-domain neuropsychological test battery, a global deficit score (GDS) and HAND diagnoses were determined for each participant. The correlation between CD4 nadir (the lowest ever lymphocyte CD4 count) and cortical thickness was investigated using a vertex-wise non-parametric approach with a conservative statistical threshold of p < 0.05 (FWE-corrected). RESULTS/ANTICIPATED RESULTS: Out of the 59 participants, 12 met standard Frascati criteria for asymptomatic neurocognitive impairment (ANI) and two met the criteria for mild neurocognitive disorder (MND). Across all participants, low CD4 nadir was associated with widespread cortical thinning, especially in the frontal and temporal regions. Higher GDS (indicating worse global neurocognitive function) was associated with bilateral frontal cortical thinning, and the association largely persisted in the subset of participants who did not meet HAND criteria. DISCUSSION/SIGNIFICANCE OF IMPACT: These results suggest that the low CD4 nadir may be associated with widespread neural injury in the brain, especially in the frontal and temporal regions. This spatial profile might contribute to the prevalence/phenotypes of HAND in the cART era, such as the frequently observed deficits in the executive domain.

